# Bacterial Viability in Self-Healing Concrete: A Case Study of Non-Ureolytic *Bacillus* Species

**DOI:** 10.3390/microorganisms11102402

**Published:** 2023-09-26

**Authors:** Augusta Ivaškė, Viktor Gribniak, Ronaldas Jakubovskis, Jaunius Urbonavičius

**Affiliations:** 1 Department of Chemistry and Bioengineering, Faculty of Fundamental Sciences, Vilnius Gediminas Technical University (VILNIUS TECH), Saulėtekio al. 11, 10223 Vilnius, Lithuania; augusta.ivaske@vilniustech.lt; 2 Laboratory of Innovative Building Structures, Faculty of Civil Engineering, Vilnius Gediminas Technical University (VILNIUS TECH), Saulėtekio al. 11, 10223 Vilnius, Lithuania; viktor.gribniak@vilniustech.lt (V.G.); ronaldas.jakubovskis@vilniustech.lt (R.J.)

**Keywords:** bacterial self-healing, cement mortar, microencapsulation, calcium alginate, viability

## Abstract

Cracking is an inevitable feature of concrete, typically leading to corrosion of the embedded steel reinforcement and massive deterioration because of the freezing–thawing cycles. Different means have been proposed to increase the serviceability performance of cracked concrete structures. This case study deals with bacteria encapsulated in cementitious materials to “heal” cracks. Such a biological self-healing system requires preserving the bacteria’s viability in the cement matrix. Many embedded bacterial spores are damaged during concrete curing, drastically reducing efficiency. This study investigates the viability of commonly used non-ureolytic bacterial spores when immobilized in calcium alginate microcapsules within self-healing cementitious composites. Three *Bacillus* species were used in this study, i.e., *B. pseudofirmus*, *B. cohnii*, and *B. halodurans*. *B. pseudofirmus* demonstrated the best mineralization activity; a sufficient number of bacterial spores remained viable after the encapsulation. *B. pseudofirmus* and *B. halodurans* spores retained the highest viability after incorporating the microcapsules into the cement paste, while *B. halodurans* spores retained the highest viability in the mortar. Cracks with a width of about 0.13 mm were filled with bacterial calcium carbonate within 14 to 28 days, depending on the type of bacteria. Larger cracks were not healed entirely. *B. pseudofirmus* had the highest efficiency, with a healing coefficient of 0.497 after 56 days. This study also revealed the essential role of the cement hydration temperature on bacterial viability. Thus, further studies should optimize the content of bacteria and nutrients in the microcapsule structure.

## 1. Introduction

Concrete has become the world’s most widely used building material because of its high compressive strength, local materials, and easy casting. However, one of the inevitable problems of concrete is crack formation. The cracks do not significantly affect the mechanical performance of concrete, but they stimulate the diffusion of water, oxygen, and various ions into the concrete. These aggressive ions reduce the concrete’s alkaline environment and cause steel reinforcement corrosion, thereby reducing the durability of the concrete structures [1,2]. Several methods of filling cracks are being investigated [3,4,5,6,7]. Different materials, such as expanding materials, chemicals, and microorganisms, are used for this purpose. 

Biological self-healing concrete is based on microorganisms-induced calcite precipitation. In nature, many bacteria could precipitate calcium carbonate. However, bacteria must survive the harsh conditions of concrete. Therefore, alkali-tolerant and spore-forming bacteria are most commonly used to produce biological concrete [8]. Three main kinds of bacteria can precipitate calcium carbonate: ureolytic, non-ureolytic, and denitrifying. Dissimilation of nitrates in the nitrogen cycle was used to produce calcium carbonate crystals in self-healing concrete by nitrate-reducing bacteria that can operate in oxygen-limited environments. Typical strains of denitrifying bacteria used for this purpose are *Pseudomonas aeruginosa* and *Diaphobacter nitroreducens* [8]. The ureolytic bacteria used to precipitate calcium carbonate produce the enzyme urease. This enzyme catalyzes the decomposition of urea to carbonate ions and the ammonia byproduct. Typically, strains of the *Bacillus* genus, such as *B. pasteurii*, *B. cereus*, *B. subtilis*, *B. sphaericus*, and *B. megaterium*, are used [9,10]. The disadvantage of bacteria that use this pathway is that although a large amount of calcium carbonate is precipitated, ammonia is released into the environment, increasing the reinforcement-corrosion risk [11,12]. To overcome the problems of urea hydrolysis, another source of nutrients was identified. Non-ureolytic bacteria can transform calcium-containing organic acids to calcium carbonate without releasing the ammonium ions. Typical alkaliphilic bacteria capable of precipitating calcium carbonate also belong to the *Bacillus* genus. The most commonly used species for research on self-healing concrete are *B. pseudofirmus*, *B. cohnii*, *B. halodurans* [12,13], *B. subtilis* [14,15], and *B. alkalinitrilicus* [16]. B. *cohnii* has great mineralization activity; moreover, all the precipitations are pure calcite [17]. In an alkaline environment, organic compounds are decomposed into CO_2_ and H_2_O, then CO_2_ is converted into ${\mathrm{CO}}_{3}^{2-}$ ions, and in the presence of Ca^2+^ ions, CaCO_3_ crystals can form because of non-ureolytic bacteria metabolism:

CaC_6_H_10_O_6_ + 6O_2_ → CaCO_3_ + 5CO_2_ + 5H_2_O (1)

The biological self-healing effectiveness relates to the long-term bacterial viability in concrete. First, during the hydration process of the concrete, the pH is extremely high, resulting in low bacterial survival in the cement matrix. Second, bacteria must survive mechanical stress during concrete mixing and hardening. Moreover, the viability of bacteria in the cementitious matrix also depends on the chemical composition of the concrete. Zinc oxide has antibacterial activity in cement admixtures, drastically reducing bacterial viability in concrete. Also, the recovery technique that involves crushing and pulverizing the cement paste could influence the viability of bacterial spores [18]. The viability of bacteria embedded directly in concrete after nine days is 1% and decreases with increasing sample age [11]. Thus, the bacteria are immobilized on various carriers to increase bacterial survival. The typical protection materials are expanded clay, perlite, diatomaceous earth, and hydrogel [19]. Expanded clay and perlite are porous materials. Bacteria and food materials are impregnated into these materials using a vacuum [20,21]. In addition, coating the surface of expanded clay (EC) particles with styrene acrylate or MgO-based layers can improve bacterial viability by approximately ten times [22]. Another promising carrier is recycled concrete aggregates (RCAs), which can replace natural resources in the production of self-healing concrete [23]. Moreover, commercial air-entraining admixtures have been proven to be an efficient alternative for protecting endospores. The viability of *B. cohnii* was maintained for nine months using this carrier [24].

Hydrogel is another promising carrier used to protect bacteria in bio-self-healing concrete. For this purpose, the natural polysaccharide alginate is applicable. Alginate is a negatively charged and long molecule composed of β-D-mannuronic acid and α-L-guluronic acid residues. Gelation reactions occur when positively charged sodium ions (Na^+^) dissolve in the solution and separate from the alginate. Then, positively charged calcium ions (Ca^2+^) can bind two different alginate chains, thus cross-linking the solution and making it thicker [25]. One of the advantages of using alginate for encapsulation is that the gelation process takes place under mild conditions [26]. For this reaction, high temperature and chemical cross-linking agents are not required [25]. After immobilizing *B. subtilis* in sodium alginate capsules, a 16% increase in compressive strength in concrete after 28 days was achieved [27]. Methacrylate modified-alginate-based hydrogel was compatible with *Bacillus sphaericus* bacteria and concrete [21]. Traditional hydrogel beads used in biologically self-healing concrete can be enhanced using chitosan. Adding 1% chitosan to calcium alginate beads increased the compressive strength by 10% and flexural strength by 14% compared to the control specimens without chitosan [28]. However, such a marginal improvement in mechanical performance describes a secondary effect. In contrast, the improved integrity of the cracked concrete structure against aggressive chemical penetration describes the main target of the healing process [3].

The laboratory tests were conducted at 20 °C and on small samples, so conditions were usually optimal for bacterial growth. However, the higher temperatures in the environment may affect bacteria viability and crystal formation in self-healing concrete. Due to high saturation, calcium carbonate crystals form faster at higher temperatures, up to 40 °C. The temperature also affects the crystal quality and morphology [29,30]. However, cement hydration involves exothermic reactions. The previous studies by the authors have shown that different cement types have distant hydration temperatures ranging from 76.5 to 88.1 °C in small cement paste specimens. Expanded clay as a carrier resulted in a more than 10-fold decrease in bacterial viability in concrete samples cured at 80 °C [18]. It is essential to investigate the potential of calcium alginate as a carrier to maintain bacterial viability at high temperatures, to enable bio-self-healing concrete with bacteria encapsulated in calcium alginate, produce large structures, and ensure more efficient crystal formation.

This study investigates the ability of calcium alginate microcapsules as a carrier to protect bacteria in cement mortar. It employs non-ureolytic *Bacillus* species to avoid ammonia release. Estimating the viability of encapsulated spores in the cementitious matrix during mixing and hardening is essential. Therefore, the research subjects are the long-term survival of bacterial spores immobilized in calcium alginate microcapsules in the mortar and the dependence of bacterial survival on the curing temperature of the samples.

## 2. Materials and Methods

### 2.1. Preparation of Spore Suspension

Three alkaliphilic and spore-forming bacteria species, i.e., *B. pseudofirmus* (DSM 8715), *B. cohnii* (DSM 6307), and *B. halodurans* (DSM 497), were purchased from the German Collection of Microorganisms and Cell Cultures (DSMZ), Braunschweig, Germany. Bacteria strains were cultivated in alkaline nutrient media containing 5 g/L peptone, 3 g/L meat extract, 0.42 g/L NaHCO_3_, and 0.53 g/L Na_2_CO_3_. The inoculated media was incubated at 30 °C temperature with shaking overnight. To obtain spore suspension, overnight culture of bacteria was inoculated to the sporulation medium containing 3.5 g/L sucrose, 4 g/L yeast extract, 0.02 g/L KH_2_PO_4_, 0.166 g/L CaCl_2_, 0.476 g/L KCl, 0.2 g/L MgSO_4_·7H_2_O, 0.2 g/L MnSO_4_·H_2_O, 4.2 g/L Na_2_CO_3_ and 5.3 g/L NaHCO_3_ [17]. Inoculated media was incubated at 30 °C temperature for four days. Bacteria spores were harvested by centrifugation (5000× *g*, 30 min), and the suspension was washed three times with sterile 10 mM Tris-HCl buffer (pH 9.0) to remove cell debris. Next, the cell suspension was heated for 30 min at 80 °C to eliminate the vegetative cells. After heating, the spore suspension was washed thrice with sterile 10 mM Tris-HCl buffer (pH 9.0). The spore suspension was quantified by staining by the Shaeffer–Fulton method. The resulting spore suspension was stored at 4 °C temperature for further use. 

To determine the number of bacterial spores in the suspension, it was serially diluted with sterile 10 mM Tris-HCl buffer (pH 9.0) to obtain 10^2^ bacterial spores/mL. For colony forming unit (CFU) estimation, dilutions of 10^3^ and 10^2^ bacterial spores/mL were spread on Petri dishes with an alkaline nutrient medium. After incubation at 30 °C overnight, the colonies were counted. The following formula determines the number of bacterial spores:

(2) $$Number\;of\;bacterial\;spores=counted\;colonies\cdot \frac{1}{dilution\;factor},$$

### 2.2. Encapsulation of Healing Agent into the Hydrogel

The encapsulation procedure was used to obtain capsules containing bacteria spores and nutrients. The spore suspension was added to the sterile encapsulation solution of 2% sodium alginate and nutrients (5 g/L peptone and 3 g/L meat extract). The encapsulation mixture’s final concentration of bacterial spores was 10^7^ bacterial spores/mL. Capsules with nutrients and without bacteria spores were used as a control. 

The mixture of the nutrients and spore solution was added dropwise to a sterile 0.2 M calcium lactate pentahydrate solution under constant stirring. The obtained capsules were allowed to stir for 30 min for full cross-linking. After that, capsules were separated from calcium lactate pentahydrate by filtering capsules through sterile filter paper and washed with clean water. Capsules were dried at 55 °C until they were dehydrated. The entire encapsulation process was performed in a laminar airflow box.

### 2.3. Viability of Encapsulated Bacterial Spores

The viability of encapsulated bacteria spores was determined by dissolving five capsules in 5 mL of the sterile citrate buffer consisting of 55 mM trisodium citrate, 30 mM anhydrous EDTA, and 150 mM NaCl (pH 8) for 30 min [26]. The samples were serially diluted in sterile 10 mM Tris-HCl buffer (pH 9). For CFU estimation, the aliquots of suitable dilutions were spread on alkaline nutrient agar plates. The colonies were counted after 24 h incubation at 30 °C temperature overnight. The number of bacterial spores per capsule was calculated. 

### 2.4. Determination of the Permeability of Nutrients Encapsulated in Hydrogel

A substance permeability study of different concentrations (1%, 1.5%, and 2%) of sodium alginate capsules with equal amounts of nutrients (5 g/L peptone and 3 g/L meat extract) was performed. Capsules without nutrients were used as a control. An appropriate number of capsules (150) was suspended in a 10% cement filtrate solution and incubated at 20 °C under strict temperature control. After 1, 7, and 14 days, samples were spectrophotometrically analyzed at 280 nm wavelength. The experiment was carried out three times.

### 2.5. Preparation of Cement Paste and Mortars

Cylindrical ∅45 × 30 mm specimens were prepared from cement paste and mortar using white CEM- I type Portland cement (Aalborg White^®^, Aalborg, Denmark), sand (0/4 mm), tap water, and obtained hydrogel capsules. Based on previous studies, white CEM-I type Portland cement does not contain metal ions with antibacterial activity, namely ZnO and CuO, which increases the survival rate of bacteria in concrete [18]. The cement paste and mortar mix proportions in Table 1 were adapted from [31]. These proportions were used to produce cement specimens. Control specimens were produced using nutrient capsules without bacterial spores. 

All the specimens were prepared in plastic cups, covered by parafilm, and left to cure for 7 days. After this period, the obtained mortar samples were used to study the bacterial viability and the effectiveness of crack healing in the cementitious matrix.

### 2.6. Encapsulated Bacterial Spores’ Viability Tests Depending on Bacteria Species

The viability of encapsulated bacterial spores in the cementitious matrix was determined 7, 14, 21, and 28 days after preparing the mortar specimens. The bacterial viability was tested on three cement paste specimens and cement mortar. During each test, a piece of the concrete sample was crushed with a hammer to release the encapsulated bacteria. For each viability test, five capsules from the cement paste or cement mortar were incubated in 5 mL of the sterile citrate buffer consisting of 55 mM trisodium citrate, 30 mM anhydrous EDTA, and 150 mM NaCl (pH 8) for 30 min. The obtained samples were serially diluted in sterile 10 mM Tris-HCl buffer (pH 9). For CFU estimation, the aliquots of suitable dilutions were spread on alkaline nutrient agar plates. The colonies were counted after 24 h incubation at 30 °C temperature overnight. The number of bacterial spores in the specimen was calculated by multiplying the number of colonies grown by the 1/dilution factor. 

### 2.7. Encapsulated Bacterial Spores’ Viability Tests Depending on the Curing Temperature

Prepared cement mortar samples were covered with a parafilm and left to set. After 24 h, they were poured with water and put into the heating chambers at different temperatures (20, 40, and 80 °C) for three days. After this period, the specimens were removed from the heating chamber and stored at 20 °C and 55 ± 5% relative humidity. The viability of encapsulated bacterial spores in the cementitious matrix depending on curing temperature was determined at 7, 14, 21, and 28 days. The bacterial viability was verified on three specimens of each curing temperature. For each viability test, five capsules from the cement paste or cement mortar were incubated in 5 mL of the sterile citrate buffer consisting of 55 mM trisodium citrate, 30 mM anhydrous EDTA, and 150 mM NaCl (pH 8) for 30 min. Next, the obtained samples were serially diluted in sterile 10 mM Tris-HCl buffer (pH 9). For CFU estimation, the aliquots of suitable dilutions were spread on alkaline nutrient agar plates. The colonies were counted after 24 h incubation at 30 °C temperature overnight. The number of bacterial spores in the specimen was calculated by multiplying the number of colonies grown using Formula (2). 

### 2.8. Quantification of Carbonization Activity

A mineralization activity assay was performed to determine the ability of bacteria to perform CaCO_3_ precipitation. The concentration of calcium ions in the solution was measured at 3, 7, and 14 days. Finally, precipitation was estimated using the previously described methodology [18]. For the mineralization activity test, media consisting of 40 mL of spore suspension (the concentration of bacterial spores in the suspension was 10^7^ CFU//mL), 5 mL of 2 M NaCl, 5 mL of inosine, 0.25 g of peptone, and 0.15 g of beef extract were kept in a rotary incubator shaker (140 rpm) at 30 °C temperature 90 min to germinate spores. Then, 50 mL of 20 mM calcium lactate pentahydrate solution was added. The concentration of calcium ions in the solution was measured at 1, 3, and 7 days. Finally, it was estimated using a calcium colorimetric assay from Sigma Aldrich. The experiment was carried out three times for each bacteria species. 

### 2.9. Crack-Healing Tests

After the curing period, three mortar samples of each bacteria species were carefully split into two parts with a hammer. The cracks were then formed by gluing the specimen parts with waterproof duct tape. The obtained specimens with cracks were immersed in tap water. The crack healing was measured after 7, 14, 21, 28, 35, 42, 49, and 56 days of recovery. Samples were dried one day before measurement. Crack healing was measured using a digital microscope with a camera (DTX 21 Levenhuk, Tampa, FL, USA). Three measuring points were chosen for each specimen. The obtained results were used to calculate the crack-healing ratio according to the following formula [16]:

(3) $$H_{c}=\frac{w_{i}-{\;w}_{t}}{w_{i}},$$

where *H_c_* is the crack-healing coefficient, *w_i_* is the initial crack width, and *w_t_* is the width of the crack after healing.

## 3. Results

### 3.1. Detection of Mineralization Activity of Bacillus Strains

**Figure 1:**
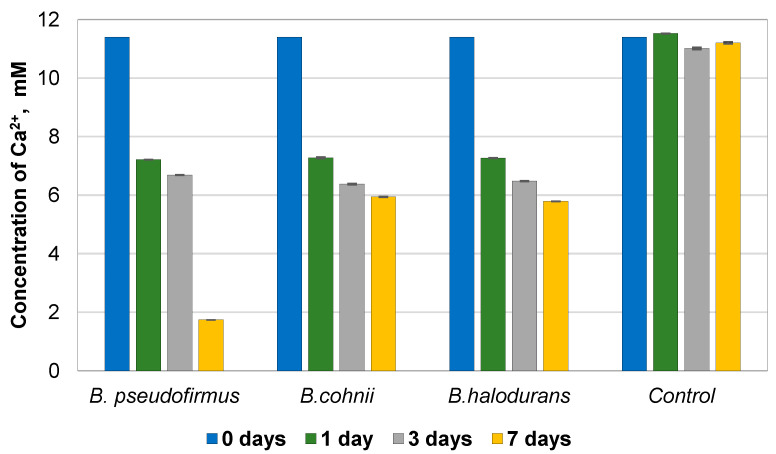
Precipitation of calcium from the medium over time by the three *Bacillus* species.

The ability of bacteria to precipitate calcium carbonate crystals is an essential process determining the healing of the cracks. For this reason, the mineralization activity of *B. pseudofirmus*, *B. cohnii*, and *B. halodurans* was investigated. Bacterial spores were activated and grown in a calcium lactate medium. After one, three, and seven days, the samples were analyzed to determine the concentration of Ca^2+^ ions in the nutrient medium. All tested bacteria species can precipitate calcium carbonate, as it is a source of Ca^2+^ in the environment. After daily incubation, the concentration of calcium ions decreased approximately 1.6 times for all *Bacillus* species used compared to the control group without bacteria (Figure 1), which suggests that bacterial spores have a function of mineralization activity after germination and supports the literature findings [17]. No significant differences were found between the mineralization activity of *B. cohnii* and *B. halodurans*. After seven days, the concentration of Ca^2+^ in the culture medium with *B. cohnii* and *B. halodurans* spores was 5.941 ± 0.009 mM and 5.789 ± 0.001 mM, respectively, while in the control group without bacterial spores, it was 11.20 mM. *B. pseudofirmus* had the best mineralization activity; after seven days, the free Ca^2+^ concentration in the culture medium was 1.742 mM. A decrease in the concentration of free calcium ions suggests that calcium carbonate is being precipitated. This ability enables its efficient application in the development of biological self-healing concrete.

### 3.2. Evaluation of Nutrient Permeability in Sodium Alginate Capsules

**Figure 2:**
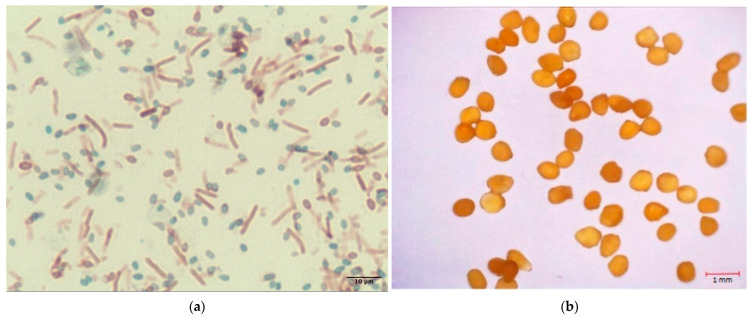
Microscopic images: (**a**) Stained *B. pseudofirmus* spores; (**b**) calcium alginate capsules containing bacterial spores.

Irregular spherical shape and a wrinkled or roughened surface, resulting from moisture loss, are characteristic of the calcium alginate capsules (Figure 2). The dimensions ranged from 0.45 mm to 0.70 mm, with an average size of 0.57 ± 0.06 mm, indicating the encapsulation method’s performance in producing carriers with a small size distribution. The encapsulation efficiency can be attributed to the viscosity of the alginate solution used for encapsulation and the method of dripping the solution, including variations in the syringe height, dripping speed, and the needle diameter used [32].

**Figure 3:**
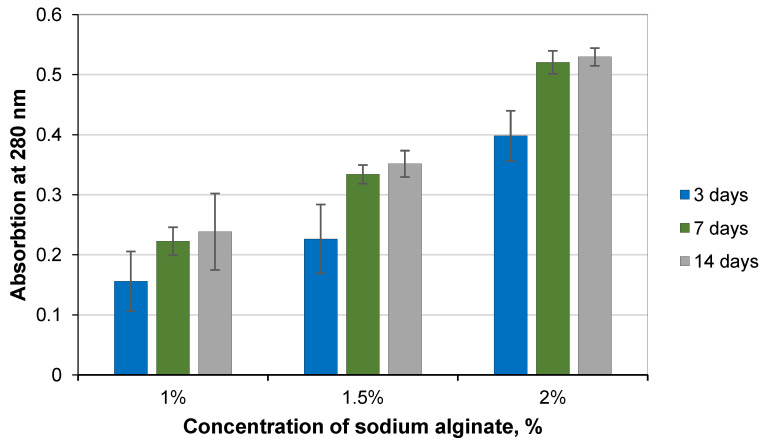
Nutrient permeability in calcium alginate capsules (triple tests; a sample = 150 capsules).

Bacteria-induced precipitation of calcium carbonate during crack formation depends on many factors. To activate bacteria in the cementitious matrix, they inevitably need nutrients not abundant in this unfavorable alkaline environment. For this purpose, additional nutrients such as yeast extract and peptone are added to the bacterial spore encapsulation mixture. Selecting the appropriate preservative concentration is essential to reduce the likelihood of nutrients leaching from the capsule matrix. Capsules with different sodium alginate concentrations (1%, 1.5%, and 2%) were stored in a cement filtration solution and analyzed by measuring light absorption at 280 nm and compared with nutrient-free control groups (Figure 3). More nutrients were released into the environment at increasing polymer concentrations. The highest diffusion of the substances was found in the capsule with a concentration of 2% sodium alginate and the lowest of 1% sodium alginate. In all study groups, the maximum release of nutrients in cement filtrate solution was found after 3 days. After 7 days, a decrease in nutrient diffusion from the capsules was observed, which almost stabilized 14 days after the start of the experiment. This indicates that releasing nutrients from carriers occurs under concentrations of nutrients in the capsule, and the solution reaches an equilibrium that aligns with the literature results [33].

As the sodium alginate concentration increases, Ca^2+^ ions can bind more alginate molecules, resulting in a more compact gel membrane and refined pore structure. As a result, the desired substance can be more embedded in the polymer matrix [34]. Although the test results show that more nutrients diffuse into the solution at higher concentrations of sodium alginate, the diffusion can be related to the higher amount of sodium alginate. It makes a higher concentration of the sodium alginate solution more suitable for further work.

### 3.3. Survival of Encapsulated Bacillus Species in the Cementitious Matrix

**Figure 4:**
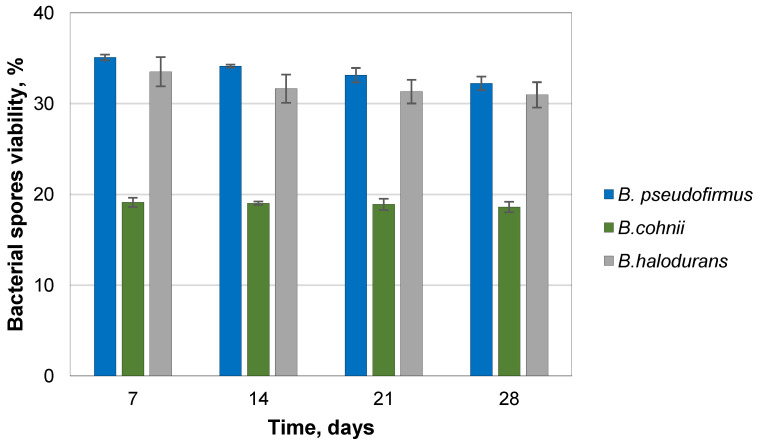
Viability of *Bacillus* species in cement paste specimens (the average of three samples).

**Figure 5:**
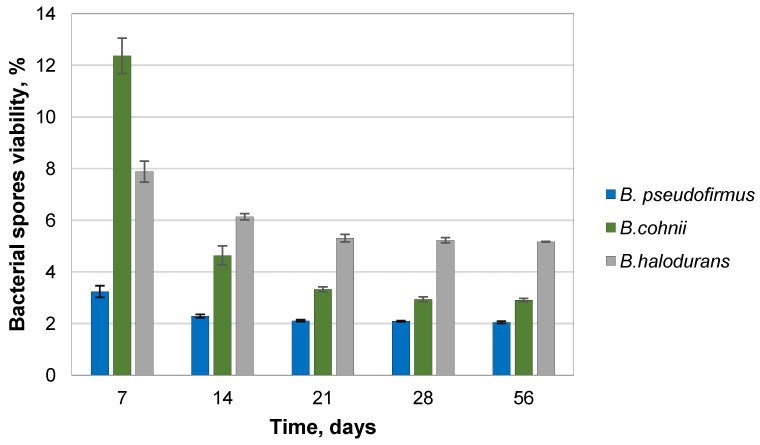
Viability of *Bacillus* species in cement mortar specimens (the average from three samples).

Spores encapsulated in 2% sodium alginate hydrogel with nutrients were used to prepare cement paste specimens, determining the bacterial ability to survive in the harsh alkaline environment. Encapsulation of the bacterial spores in calcium alginate produced microcapsules with spore concentrations of *B. pseudofirmus*, *B. cohnii*, and *B. halodurans* in capsules of 2.52 × 10^7^ CFU/capsule, 1.27 × 10^7^ CFU/capsule, and 9.17 × 10^7^ CFU/capsule, respectively. These calcium alginate beads were used for the viability studies. The viability of encapsulated spores embedded in the mortar samples was significantly reduced at the beginning of the experiment in all study groups (Figure 4). To check bacterial viability, two different dilutions were made for each sample, and each dilution was spread in three Petri dishes. The most significant adverse effect was observed for *B. pseudofirmus* and *B. halodurans* bacteria, where viability remained relatively constant at 32 ± 0.74% and 31 ± 1.40% at day 28, respectively. The lowest viability of encapsulated bacteria in cement paste was found for *B. cohnii* bacteria. It did not change during the investigation and accounted for 19% of the initial spore viability. No bacterial growth was observed in control groups without bacterial spores. Then, capsules with bacterial spores were incorporated into the cement mortar. The most significant adverse effect was for *B. pseudofirmus* bacteria, where the viability after 7 days was 3.24 ± 0.23%. After 14 days of curing, it was 2.29 ± 0.07% and remained stable throughout the experiment (Figure 5). Two dilutions were made for each sample. Each dilution was spread in three Petri dishes. Although *B. cohnii* had the highest viability percentage at the beginning of the experiment (12.36 ± 0.69%), it decreased to 2.90 ± 0.07% after 56 days. The viability of *B. halodurans* exceeded 5% during the whole period. No bacterial growth was observed in the control groups. The Portland cement was not sterilized, but as it has a pH of 10–12, it is a harsh environment for the bacteria. Only alkaliphilic bacteria encapsulated in carriers can survive such conditions.

### 3.4. The Curing-Temperature Effect on Bacterial Viability

**Figure 6:**
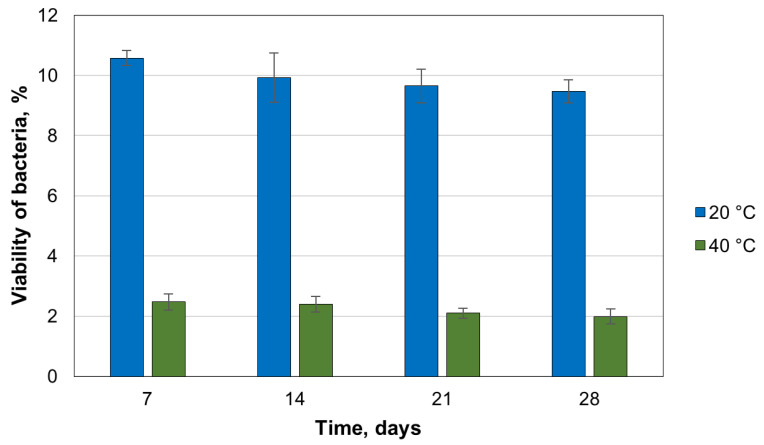
Viability of *B. pseudofirmus* in the mortar specimens depending on the curing temperature (the average values from three samples).

The mortar production process generates high temperatures during cement hydration. Therefore, this study evaluates the impact of the curing temperature of the samples on the viability of *B. pseudofirmus* spores. This bacterial species was chosen because of its stable viability in cement mortar specimens. Viability was studied at 20 °C, 40 °C, and 80 °C. The results show that 2.1% (1.51 × 10^5^ CFU/capsule) of the encapsulated bacterial spores were recovered after 28 days of the mortar cured at 40 °C compared to the control specimens cured at 20 °C (Figure 6). However, the viability drastically decreased in the mortar samples cured at 80 °C. After 7 days, the number of viable bacterial spores was undetectable. 

### 3.5. Evaluating the Crack-Healing Efficiency

**Figure 7:**
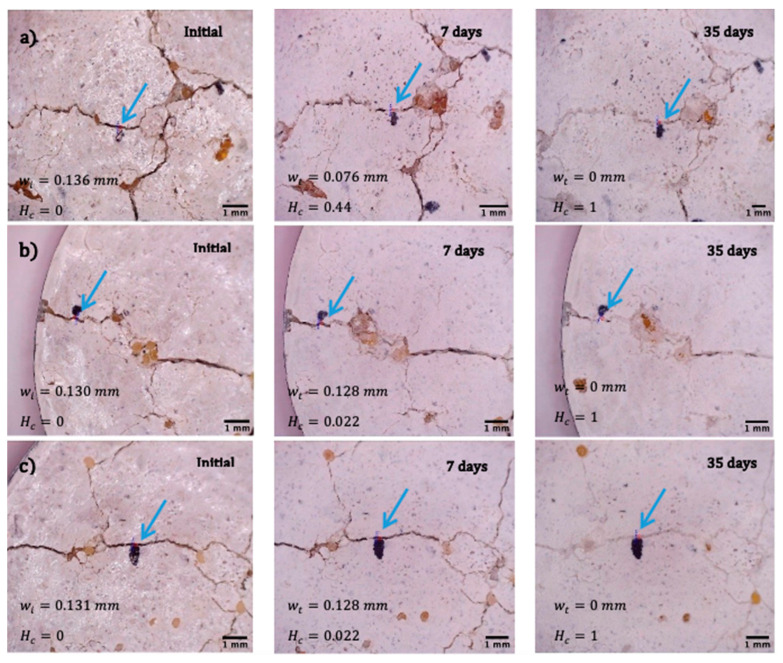
Microscopic images (ranging in magnification from 24× to 28×) of mortar samples with a crack width of 0.130-0.136 mm before and after 7 and 35 days of healing using microcapsules with different bacterial species: (**a**) *B. pseudofirmus*; (**b**) *B. cohnii*; (**c**) *B. halodurans.* Here, *w_i_* is the initial crack width; *w_t_* is the crack width after healing; *H_c_* is the calculated healing ratio. The blue arrows indicate the location where the width of the gap was measured.

**Figure 8:**
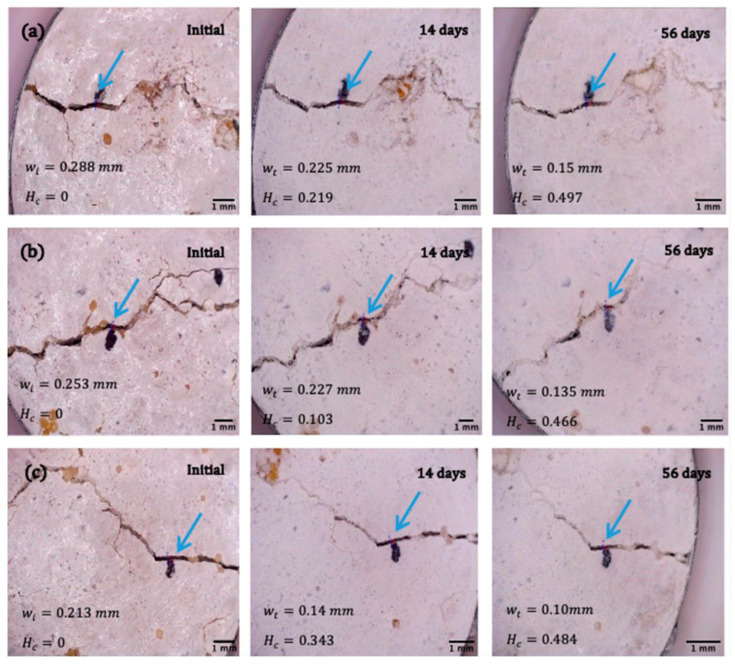
Microscopic images (ranging in magnification from 24× to 28×) of mortar samples with a crack width of 0.213-0.288 mm before and after 14 and 35 days of healing using microcapsules with different bacterial species: (**a**) *B. pseudofirmus*; (**b**) *B. cohnii*; (**c**) *B*. *halodurans.* Here, *w_i_* is the initial crack width; *w_t_* is the crack width after healing; *H_c_* is the calculated healing ratio. The blue arrows indicate the location where the width of the gap was measured.

To determine the ability of encapsulated *B. pseudofirmus*, *B. cohnii*, and *B. halodurans* to heal cracks in concrete structures, cracks of different sizes were opened after 28 days in prepared mortar specimens, as described in the “Crack healing tests” section. The healing factor *H_c_* (Equation (3)), ranging from 0 (unhealed damage) to 1 (fully healed defect), evaluates the effectiveness of crack treatment. The studies described above have revealed that the viability of bacteria in mortar decreases drastically over time. To assess the efficacy of crack filling with viable bacteria, it was chosen to test the effectiveness of the crack filling 7 days after the specimens had been produced rather than waiting until the samples were fully cured. Analysis of the bacterial ability to heal 0.130–0.136 mm cracks (Figure 7) demonstrates that samples with encapsulated *B. cohnii* and *B. halodurans* bacteria had a higher healing rate—the cracks were filled after 14 days. The mortar specimens with cracks ranging from 0.213 mm to 0.288 mm (Figure 8) did not heal throughout the study. After 56 days of self-healing, the width of the crack and the self-healing coefficients were similar for samples with all bacterial species and reached 0.497, 0.466, and 0.484 for *B. pseudofirmus*, *B. cohnii*, and *B. halodurans*, respectively.

**Figure 9:**
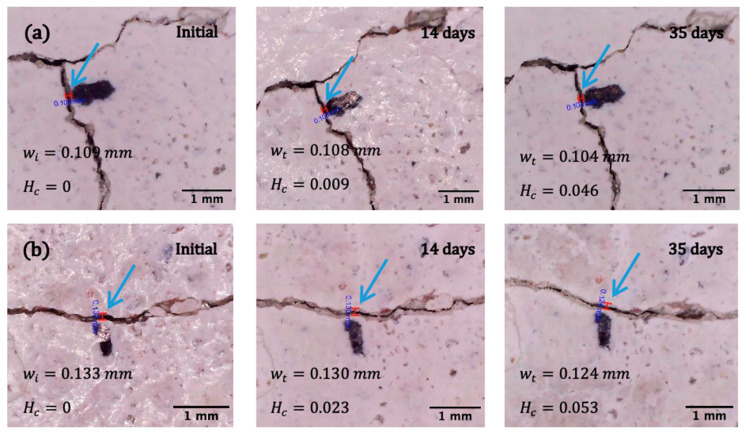
Microscopic images (ranging in magnification from 24× to 28×) of control mortar samples before and after 14 and 35 days of healing: (**a**) mortar samples without microcapsules; (**b**) mortar samples with capsules with nutrients and without bacteria spores. Here, *w_i_* is the initial crack width; *w_t_* is the crack width after healing; *H_c_* is the calculated healing ratio. The blue arrows indicate the location where the width of the gap was measured.

The study used two control samples: a sample without capsules and one with calcium alginate capsules without bacterial spores and with nutrients only. The cracks were not filled (Figure 9). Thus, the healing coefficient does not exceed 0.2 in all tested control samples. This result reveals the autogenous healing property of the concrete manifested in the ability to close small cracks without adding a healing agent [35]. 

**Figure 10:**
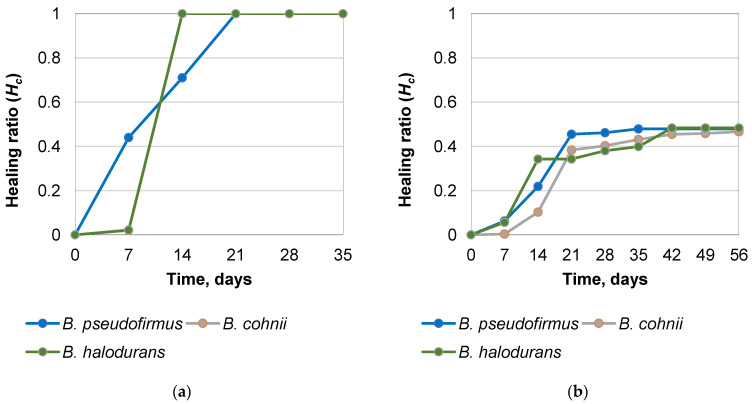
Time-dependence of self-healing ratio in mortar samples using different bacterial species: (**a**) with a crack width of 0.130–0.136 mm; (**b**) with a crack width of 0.213–0.288 mm.

Figure 10 summarizes the recovery results. In the case of wider slits with a width of 0.288 mm, the healing coefficient is almost the same for all tested bacterial species. However, *B. pseudofirmus* shows the highest healing rate. 

During the study, the samples were stored in water. The pH of the water was measured weekly and varied from 8.4 to 9.7, ensuring favorable and uniform environmental conditions for bacterial activity and CaCO_3_ formation, which allowed an accurate assessment of the dependence of crack healing on the bacterial species. However, these conditions are non-real; thus, field tests must verify the observed results [3].

## 4. Discussion

The study investigated the viability of *Bacillus* species in a cement paste and mortar. *Bacillus* is an alkaliphilic bacteria suitable for use in biological concrete. The study showed that the number of spores of *B. pseudofirmus*, *B. cohnii*, and *B. halodurans* does not decrease after encapsulation in calcium alginate microcapsules. In previous studies, it was found that when *B. lichemiformis* bacteria were encapsulated in calcium alginate, their viability after encapsulation reached 99% [36]. Calcium alginate capsules are a suitable carrier for encapsulating bacteria in self-healing concrete. The most significant adverse effect on bacterial viability was found in the mortar, which has a much more rigid structure than the cement paste. Hence, the capsules were under higher pressure. In addition, they were exposed to the harsh alkaline environment’s harmful effects during the samples’ curing. The study also shows that the bacterial spores’ encapsulation in 2% sodium alginate reduces the initial viability from 10 to 100 times. This may be due to too low a concentration of polymer or bacteria used. Nevertheless, the survival rate and survival time are higher for those incorporated directly into the cement mortar. These results align with the study of bacterial concrete [33], which demonstrated that *B. pseudofirmus* encapsulated in calcium alginate microcapsules remained viable after concrete mixing. Most studies have investigated the self-healing performance of bio-concrete without considering the long-term bacterial viability of the cement matrix. They assess the mechanical properties of the concrete, such as compressive strength and water permeability, after self-healing. Thus, this study focuses on long-term bacterial viability in the cement matrix and viability under elevated temperatures during cement hydration.

This study determined bacterial viability by counting the number of colonies grown on a solid nutrient medium. Meanwhile, the viability of the ureolytic bacteria could be determined indirectly by measuring the activity of the enzyme urease as the concentration of NH_3_ released by the encapsulated bacteria changes in the environment. This method applies only to such bacteria and cannot be used to determine the viability of non-ureolytic bacteria in the cement matrix. The viability of non-ureolytic bacteria is measured by the direct dilution-inoculation method, where the bacteria are spread on Petri plates, and the grown colonies are counted. The viability can be determined precisely because the required number of capsules is removed from the concrete sample and dissolved in a mixture of Na-citrate and EDTA using calcium alginate microcapsules. Combining these substances debonds calcium alginate by chelating the bond between calcium ions and alginate fibers. The combination of these substances has been found to have no effect on bacterial spore viability and can be used to test bacterial viability [33]. However, the limitation of the direct dilution-inoculation method is that some bacterial cells can be metabolically active but cannot divide. Meanwhile, directly determining bacterial viability using expanded materials is barely possible. In this case, the concrete samples are broken and crushed, so the number of bacteria is affected by how much curing agent enters the test sample [22].

Bacterial viability drastically decreased when mortar specimens were cured at 80 °C. These results align with the previous findings of bacterial spores encapsulated in expanded clay [18] and suggest that encapsulation to the hydrogel technology cannot protect bacteria from the effect of high temperatures. This finding proclaims the necessity of additional means for reducing the cement hydration-induced heating temperature and defines the object for further research. Proper protection of bacterial spores encapsulated in calcium alginate from the influence of high temperatures can increase their viability, and accelerate the formation and improve the quality of calcium carbonate crystals [14,15]. 

*B. pseudofirmus* showed the highest efficiency of crack healing. Small (0.130–0.36 mm) cracks were filled within 14 days. This observation aligns with the results that demonstrated the viability of *B. pseudofirmus* bacteria encapsulated in calcium alginate and the ability of these bacteria to fill relatively small defects within 56 days [33]. However, the larger cracks of 0.213–0.288 mm were not filled. In contrast, Gao et al. reported that, encapsulated in calcium alginate with chitosan, *B. pseudofirmus* bacteria filled 1 mm cracks [37]. Incomplete and uneven crack healing can result from insufficient bacterial viability and protection of the polymeric coating, an inferior self-healing agent amount, and uneven distribution in the samples. To increase the viability of the bacterial spores in the cementitious matrix and to heal wider cracks, the composition of the microcapsules should be adjusted by increasing the concentration of bacteria and the nutrient content. In addition, coatings could protect the microcapsules from a harsh alkaline environment and elevated cement hydration temperatures. At the same time, the observed crack closure provides only indirect evidence of bacterial activity. Thus, bacteria identification and quantification in realistic environmental conditions describe a further research object.

Future research should follow several directions. First, conditions for bacterial survival in the cement matrix should be improved. The resulting hydrogel capsules should be coated or impregnated to increase the survival of bacteria in the cement matrix. This would increase the self-healing efficiency of the concrete to fill the broadest possible crack widths in the shortest possible time. Another area for further research should be related to studies under natural environmental conditions. To date, most of the research on bio-concrete has been carried out to optimize the composition of bio-concrete and to test its mechanical properties under laboratory conditions. The performance of bio-self-healing concrete needs to be tested under realistic outdoor conditions where the concrete is exposed to temperature, humidity, and freeze–thaw cycles.

## 5. Conclusions

This study investigated the effect of cement hydration temperature on the viability of *Bacillus* species encapsulated in calcium alginate microcapsules in a cement matrix. The investigation results demonstrate that calcium alginate microcapsules are promising carriers for bacteria in self-healing concrete, protecting the bacteria’s viability during the cement hydration period. It was observed that *B. pseudofirmus* and *B. halodurans* spores had the highest viability after incorporating the microcapsules into the cement paste, while *B. halodurans* spores retained the highest viability in the cement mortar during 56 days of monitoring. At the same time, it was found that a temperature exceeding 80 °C makes the bacteria no longer viable. Therefore, bacterial healing should be used with particular care when the cement hydration temperature can exceed the above boundary, e.g., in massive concrete structures.

Mortar has a stiff internal structure compared to the cement paste; therefore, the capsules are exposed to more significant pressure and the adverse effects of a harsh environment during the curing process. Thus, the viability of *Bacillus* species in mortar is significantly lower than in cement paste.

The fastest filling of cracks in the mortar was achieved with *B. halodurans* and *B. cohnii* bacteria species at a crack width of about 0.13 mm. However, wider cracks (up to 0.288 mm) were not healed within 56 days, demonstrating healing coefficients of 0.497, 0.466, and 0.484 for *B. pseudofirmus*, *B. cohnii*, and *B. halodurans*. The bacterial and nutrient levels should be optimized in further studies. Hence, the composition and protection of the microcapsules require improvements. Once the microcapsule production technology and upscaling have been optimized, bio-self-healing concrete could be used for bridge construction because of the complexity of alternative retrofitting techniques and limited access to deteriorated superstructures.

Taking into account the calcium carbonate precipitation activity, the viability of the bacteria in cement paste and cement mortar, and the efficiency of crack filling, *B. pseudofirmus* is the most promising species of non-ureolytic bacteria that can be encapsulated in calcium alginate microcapsules and can be used in self-healing concretes.

## Figures and Tables

**Table 1:** Compositions of cement paste and mortar (quantity for a single test sample).

Cement Mortar		Cement Paste		Material
Without Capsules	With Capsules	Without Capsules	With Capsules	
-	0.8 g	-	0.8 g	Capsules
15 g	15 g	30 g	30 g	Cement
15 g	15 g	-	-	Sand
6 g	6 g	8 g	8 g	Water

## Data Availability

Data are available from the authors upon request.
